# Retinal Toxicity after Initial Administration of Nivolumab and Ipilimumab

**DOI:** 10.1155/2023/9931794

**Published:** 2023-12-21

**Authors:** Adnan Kilani, Efstathios Vounotrypidis, Susanna F. König, Armin Wolf

**Affiliations:** Department of Ophthalmology, University Hospital Ulm, Prittwitzstraße 43, 89075 Ulm, Germany

## Abstract

**Background:**

To present a rare case of a bilateral immune checkpoint inhibitor- (ICI-) induced photoreceptor injury with a bacillary layer detachment (BALAD) and a dissection of the photoreceptor inner and outer segment, accompanied by ICI-induced Vogt-Koyanagi-Harada- (VKH-) like uveitis after initial administration of nivolumab and ipilimumab. *Case Presentation*. A 52-year-old female with metastatic malignant cutaneous melanoma experiencing bilateral progressive visual acuity reduction, after treatment initiation with 1 mg/kg nivolumab and 3 mg/kg ipilimumab two weeks prior symptom onset. An extensive laboratory workup, including uveitis workup, onconeuronal and retinal antibodies, ruled out a paraneoplastic autoimmune disorder and a granulomatous disease. Furthermore, a B-scan was performed to exclude a posterior scleritis. Ensuing temporary treatment discontinuation of nivolumab and complete discontinuation of ipilimumab, treatment with high-dose systemic steroids was initiated, which resulted in alleviation of her symptoms and stability of ocular findings.

**Conclusions:**

ICIs can induce significant ocular side effects. As ocular inflammation can be well controlled using systemic steroids, treatment with ICIs can be continued whenever possible, in particular, if there is a good treatment response of the systemic malignancy.

## 1. Introduction

Treatment of metastatic cutaneous melanoma has undergone rapid advances with the development and clinical application of immune checkpoint inhibitors (ICIs). Significantly improved clinical outcomes can be seen in patients with metastatic melanoma and other cancer entities under treatment with ICIs [1, 2]. Although ICIs provide effective treatment, some cases are associated with ocular adverse events [1, 2]. There have been several case reports documenting occurrences of uveitis in patients taking nivolumab and other PD-1 inhibitors [3–6]. Other adverse ocular effects associated with immunotherapy include retinopathy, clinical myasthenia gravis, and orbital inflammation [5].

Our reported case involved a female patient with malignant cutaneous melanoma being treated with a combination immunotherapy, thus demonstrating a substantially higher risk for an ICI-related ocular inflammation compared to patients with nonmelanoma cancer [2]. In this context, we report a case of bilateral immune-mediated retinopathy in a patient undergoing a combination immunotherapy with ipilimumab and nivolumab for metastatic cutaneous melanoma.

## 2. Case Presentation

A 52-year-old female Caucasian presented with blurry vision and bilateral visual acuity impairment in June 2021 at the Department of Ophthalmology, University of Ulm, Germany. She complained of a progressive painless reduction in visual acuity over the last 3 weeks, with no indication of prodromal symptoms such as a cold or fever. A detailed medical history revealed the diagnosis of metastatic malignant cutaneous melanoma originating from her left upper limb approximately 2 months ago. Staging had then revealed an extensive metastasis of the cutaneous and soft tissues, involving bone tissues and multiple organs.

According to the patient, the symptoms had been occurring progressively within two weeks after the initial administration of 1 mg/kg nivolumab and 3 mg/kg ipilimumab. No previous eye surgeries or other symptoms were reported, and the only concomitant medication was ASS 100 mg and vitamin supplements.

**Figure 1:**
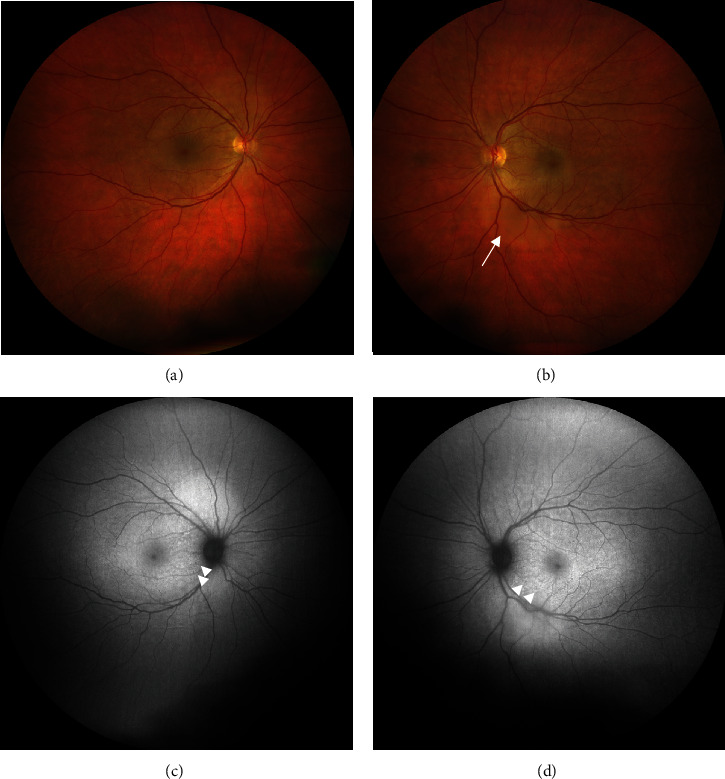
Multimodal imaging at initial presentation. (a, b) Fundus photograph of both eyes shows mild narrowing of the retinal arterial vessels and an ovoid elevation of the retina in the left eye ((b), white arrow). (c, d) Fundus autofluorescence of both eyes demonstrates small patches of hypoautofluorescence surrounded by a ring of hyperautofluorescence scattered in the macular region (white arrowheads).

**Figure 2:**
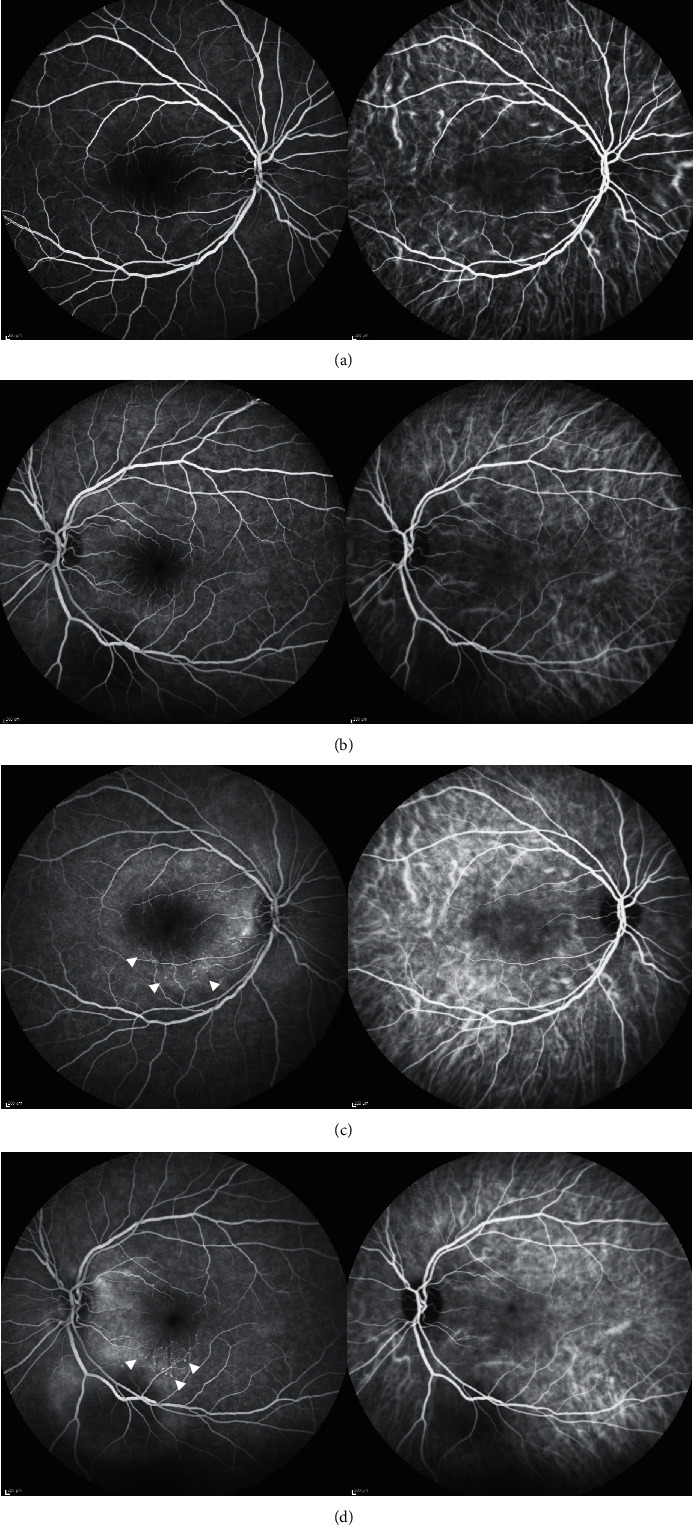
(a, b) Early phase of combined fluorescein and indocyanine angiographic examination illustrates choroidal congestion without signs of hyperpermeability or hypofluorescent areas. (c, d) Late phase of combined fluorescein and indocyanine angiographic examination shows discrete extravascular fluorescence (white arrowheads) without signs of retinal vasculitis or loss of choroidal vascular details in both eyes.

**Figure 3:**
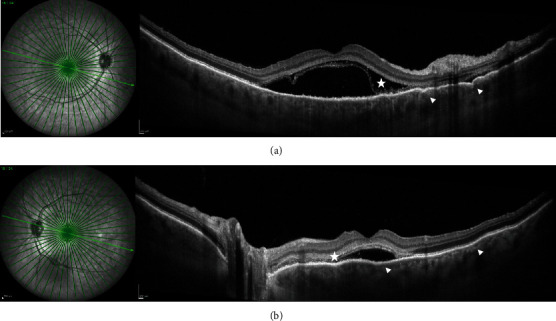
SD-OCT of the macula at initial presentation. (a, b) SD-OCT demonstrates pachychoroidal changes (choroidal thickness of 505 *μ*m in the right eye and 510 *μ*m in the left eye) with discrete choroidal folds (white arrowheads) and subretinal fluid bilaterally, typically seen in immune checkpoint inhibitor- (ICI-) induced Vogt-Koyanagi-Harada- (VKH-) like uveitis. The most distinctive finding in this OCT assessment is the bacillary layer detachment with a dissection of the photoreceptor inner and outer segment in both eyes (white star), apparently more extensive in the right eye.

Her best-corrected visual acuity (BCVA) at presentation was “counting fingers” (CF) close to the face in each eye. Intraocular pressure was 16 mmHg in both eyes. The anterior segment showed no signs of inflammation, granulomatous keratic precipitates, or posterior synechiae. Fundus examination revealed mild narrowing of the retinal arterial vessels and scarce vitreous cells (according to vitreous cell grading scale used in the Multicenter Uveitis Steroid Treatment Trial: score 0.5+) bilaterally (Figures 1(a) and 1(b)). Fundus autofluorescence presented with bilateral small patches of hypoautofluorescence surrounded by a ring of hyperautofluorescence scattered in the macular region (Figures 1(c) and 1(d)). Combined fluorescein and indocyanine angiographic examination only showed, in the early phase, choroidal congestion and, in the late phase, discrete extravascular fluorescence without signs of retinal vasculitis or loss of choroidal vascular details in both eyes (Figure 2). OCT demonstrated pachychoroidal changes (choroidal thickness of 505 *μ*m in the right eye and 510 *μ*m in the left eye) with discrete choroidal folds and subretinal fluid bilaterally, typically seen in immune checkpoint inhibitor- (ICI-) induced Vogt-Koyanagi-Harada- (VKH-) like uveitis (Figure 3). However, the most distinctive finding in the OCT assessment was the bacillary layer detachment (BALAD) with a dissection of the photoreceptor inner and outer segment in both eyes, apparently more extensive in the right eye (Figure 3).

Magnetic resonance imaging (MRI) of the head, neck, and orbits was negative for metastatic lesions, strokes, pituitary adenitis, ocular myositis, or optic neuritis. Due to the presence of multiple soft tissue metastases in the lumbar region, a cerebrospinal fluid puncture was refrained. An extensive laboratory workup, including uveitis workup, onconeuronal and retinal antibodies, ruled out a paraneoplastic autoimmune disorder and a granulomatous disease. Furthermore, a B-scan was performed to exclude a posterior scleritis.

**Figure 4:**
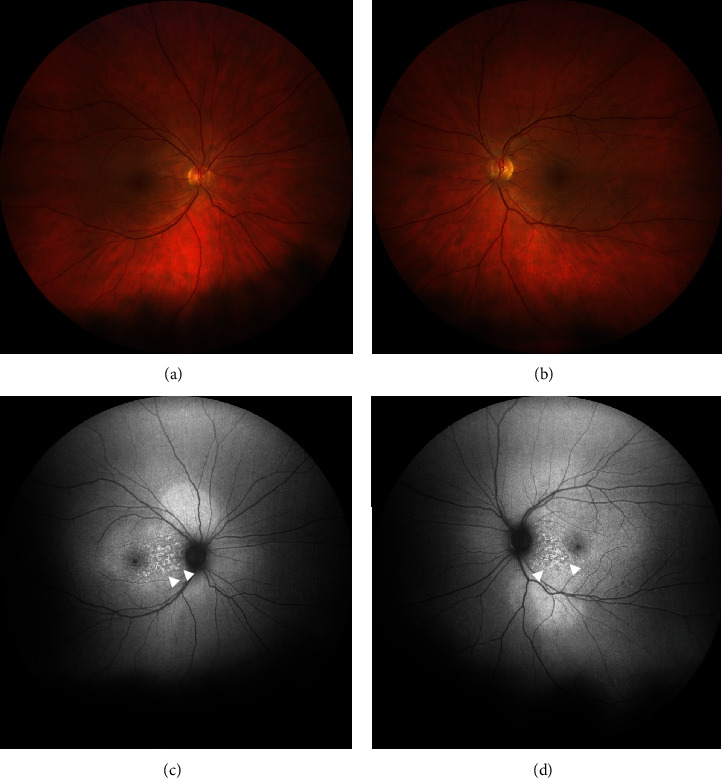
Multimodal imaging eight days after initial presentation. (a, b) Fundus photograph of both eyes shows improvement with a rapid response to methylprednisolone and complete resorption of the subretinal fluid. (c, d) Fundus autofluorescence of both eyes shows alterations with increase in small patches of hypoautofluorescence surrounded by a ring of hyperautofluorescence scattered in the macula (white arrowheads).

**Figure 5:**
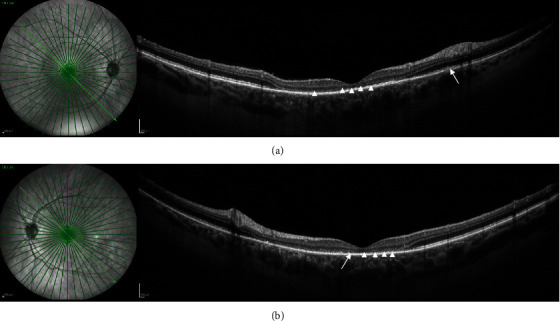
SD-OCT of the macula eight days after initial presentation. (a, b) SD-OCT demonstrates pachychoroidal changes (choroidal thickness of 495 *μ*m in the right eye and 490 *μ*m in the left eye) with discrete alterations of the retinal pigment epithelium (RPE) (white arrow) and photoreceptor damage (white arrowheads) with ellipsoid zone disruption (white arrowheads) in both eyes.

**Figure 6:**
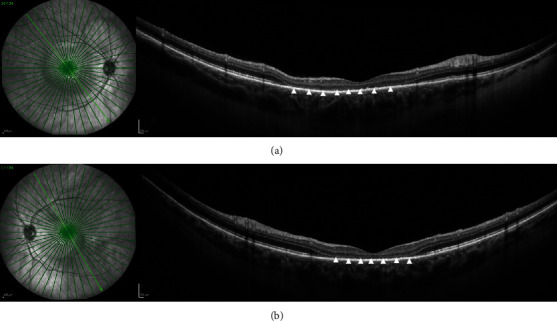
SD-OCT of the macula four weeks after initial presentation. (a, b) SD-OCT demonstrates pachychoroidal changes with photoreceptor damage (white arrowheads) and ellipsoid zone disruption (white arrowheads) in both eyes.

**Figure 7:**
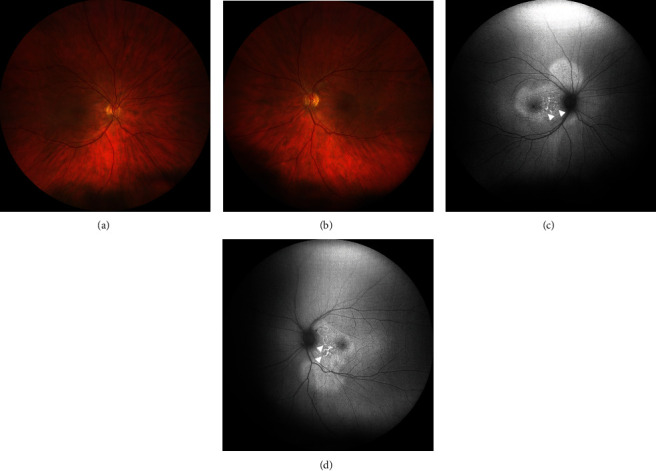
Multimodal imaging 14 weeks after initial presentation. (a, b) Fundus photograph of both eyes shows stable findings compared to clinical presentation 8 days after the initial examination. (c, d) Fundus autofluorescence of both eyes shows a decrease and consolidation of small patches of hypoautofluorescence surrounded by a ring of hyperautofluorescence scattered in the macula compared to the findings 8 days after initial presentation (white arrowheads).

**Figure 8:**
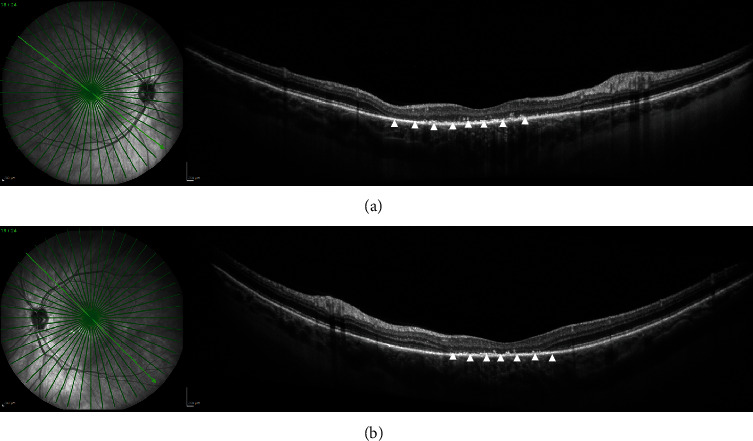
SD-OCT of the macula 14 weeks after initial presentation. (a, b) SD-OCT demonstrates pachychoroidal changes (choroidal thickness of 420 *μ*m in the right eye and 480 *μ*m in the left eye) with photoreceptor damage (white arrowheads) and ellipsoid zone disruption (white arrowheads) in both eyes.

In summary, the interdisciplinary workup and multimodal imaging of both eyes revealed suspicion of retinal toxicity with attenuation of the photoreceptor layer secondary to combined immunotherapy with nivolumab and ipilimumab. Treatment with nivolumab was temporarily stopped, ipilimumab was completely discontinued, and the patient was started on systemic steroids with intravenous methylprednisolone 1 g/daily. She showed a rapid response to cortisone therapy, with symptoms improving within two days and a marked improvement reported after five days. After intravenous methylprednisolone, the patient was switched to weight-adjusted oral cortisone (methylprednisolone 1 mg/kg/day) for four weeks, followed by tapering. After eight days (Figures 4 and 5), ocular examination revealed slight improvement of clinical findings with a BCVA of 0.7 logMAR in the right and 0.6 logMAR in the left eye. OCT-imaging pointed towards photoreceptor damage and ellipsoid zone disruption probably due to the ocular toxicity of nivolumab and ipilimumab (Figure 5). Further follow-ups were conducted after four (Figure 6) and 14 weeks (Figures 7 and 8) and demonstrated clinically stable findings with a decrease in choroidal thickness to 420 *μ*m in the right eye and to 480 *μ*m in the left eye (Figure 8), as well as unchanged BCVA with 0.6 logMAR in both eyes at the final visit.

In cooperation with the patient's oncologist, immunotherapy was reinstituted with nivolumab monotherapy without systemic steroids ten weeks after her initial ophthalmic examination.

Encouragingly, the metastatic melanoma has regressed and responded well to monotherapy with nivolumab. However, further ophthalmic assessments are essential.

## 3. Discussion

This patient with metastatic cutaneous melanoma developed a photoreceptor toxicity in conjunction with autoimmune retinopathy shortly after the initial administration of a combination immunotherapy of ipilimumab and nivolumab. Different reports have described an association between monoimmunotherapy and ocular toxicity that develops shortly after treatment initiation [4]. Furthermore, there have been multiple case reports of uveitis in patients taking nivolumab and other PD-1 inhibitors [4–6]. It occurs relatively rarely (in approximately 1%) but may be more frequent in those using more than one ICI and in female patients with malignant melanoma [2, 5, 6]. Our reported case involved a female patient with malignant cutaneous melanoma being treated with a combination immunotherapy, thus demonstrating a substantially higher risk for an ICI-related ocular inflammation compared to patients with nonmelanoma cancer [2].

Melanoma-associated retinopathy (MAR), a poorly understood condition, is a term referring to an immunologic process evoked by paraneoplastic cascades that lead to retinal antigens being detected as autoantigens by the immune system. Hence, in our case, an extensive laboratory workup, including onconeuronal and retinal antibodies (to determine the following antibodies: aquaporin-4, glutamic acid decarboxylase, recoverin, and *α*-enolase), was addressed to rule out a paraneoplastic syndrome. The patient's response to halting the immunotherapy and initiating systemic steroid treatment supports our suspected diagnosis of an immune-mediated retinopathy due to ipilimumab and nivolumab toxicity. MAR, which often requires extended immunosuppression to be under control, would have been expected to worsen, or the initial symptoms would have recurred despite halting treatment for her metastatic cutaneous melanoma and tapering off the steroids. Instead of this, the patient's symptoms improved, and her retinal lesions remained completely stable throughout the follow-up.

We provide evidence of a case of probable autoimmune retinopathy caused by ipilimumab and nivolumab in a patient with metastatic cutaneous melanoma. ICIs have revolutionized the management of several previously untreatable metastatic diseases. Since the use of this kind of medication continues to increase, one must remain vigilant in monitoring and reporting side effects. Although uncommon, ocular side effects can be serious and irreversible. Therefore, we recommend that all patients receiving immunotherapy obtain a baseline comprehensive ocular check-up, be counseled on the possible ocular side effects, and be notified to seek medical attention immediately if they experience any kind of visual changes.

## Data Availability

All data underlying the results are available as part of the article, and no additional source data are required.

## Abbreviations

BALAD: Bacillary layer detachment
BCVA: Best-corrected visual acuity
ICI: Immune checkpoint inhibitor
MAR: Melanoma-associated retinopathy
MRI: Magnetic resonance imaging
VKH: Vogt-Koyanagi-Harada.
